# A study on young male drivers’ performance and cognitive shifts under time-reduction-goal tasks

**DOI:** 10.1371/journal.pone.0335753

**Published:** 2025-11-25

**Authors:** Shuai Huang, Yanqing Yao, Zhengwu Wang, Jie Wang

**Affiliations:** 1 School of Transportation, Changsha University of Science and Technology, Changsha, China; 2 Hunan Key Laboratory of Smart Roadway and Cooperative Vehicle-Infrastructure Systems, Changsha University of Science and Technology, Changsha, China; Liaoning Normal University, CHINA

## Abstract

Drivers often engage in aggressive behaviors during time-reduction-goal tasks without fully understanding the actual time saved. This study investigated how such goals influence driving behavior and perception. A total of 99 young male drivers initially completed a survey assessing their beliefs about time-saving performance. Of these, 32 were randomly selected to participate in real driving experiments under both time-reduction and control conditions. Heart rate (HR), skin conductance response (SCR), and driving data were collected. Afterward, the experimental results were shared with all 99 drivers who completed the initial survey, including the 32 experimental participants and 67 non-participants. All drivers then provided cognitive feedback. The findings indicated that: (1) 78% of drivers believed that aggressive driving reduced both traffic light-affected time (TLT) and non-traffic light-affected time (NTLT); (2) Time-reduction goals led to more frequent acceleration and deceleration, reducing total travel time primarily in NTLT segments, while TLT remained stable. HR and SCR showed no significant increase in anxiety; (3) After receiving feedback, 72.7% of drivers, including 85.2% of participants and 69.4% of non-participants, agreed that aggressive driving had limited impact on TLT and expressed a willingness to modify their behavior. This study revealed actual behavioral outcomes under time pressure, assessed the potential of cognitive feedback, and provided insights for promoting safer and more efficient driving.

## 1. Introduction

Road traffic accidents remain a major global safety concern, resulting in high fatality rates and economic losses estimated at 10%–12% of global GDP [1]. Although many accidents appear random, studies consistently show that driver behavior is the leading cause. Crashes linked to impaired conditions—such as alcohol use, drug consumption, or fatigue—are especially devastating. In response, numerous countries have implemented laws to curb these high-risk behaviors. Among psychological factors, anxiety is particularly common and poses a serious threat to driving safety. However, anxiety is shaped by both the driving environment and task demands, making it difficult to detect and quantify in real time. This challenge in measuring anxiety’s impact on road safety remains a pressing issue for researchers [2].

Time anxiety is a prevalent type of anxiety encountered during driving tasks, frequently brought about by time-sensitive tasks like work commitments, personal engagements, or catching transportation. The presence of deadlines plays a key role in inducing psychological responses in individuals [3], and the psychological stress resulting from these time constraints is termed “time urgency” [4], which is a type of time-related stress. Research indicates that time-related anxiety can have a notable impact on cognitive functions; when under time constraints, individuals may struggle to fully process information, leading to potential inaccuracies in their judgments and decision-making abilities [5,6]. This capability is particularly crucial for ensuring driving safety, as a decrease in a driver’s capacity to get traffic-related information may result in insufficient processing of essential details such as the movements and velocities of other vehicles and road conditions, consequently elevating the risks associated with driving and, in severe instances, potentially causing traffic incidents [7].

At the same time, it has been confirmed that time anxiety can lead to dangerous driving behaviors, such as increased acceleration and deceleration rates, higher average speeds, and frequent lane changes, all of which are detrimental to road safety. Research has indicated that drivers of different occupations and driving experiences exhibit varying changes in driving behavior under time anxiety, Taxi drivers tend to exhibit higher speed increases compared to general drivers [8]. Additionally, factors such as road landscape, traffic flow, and vehicle performance play significant roles in influencing the effect of time anxiety on driving behavior. A comparative study conducted in a simulated driving environment by Fitzpatrick and others [9] demonstrated that when faced with imminent end of green light, drivers with time anxiety often choose to speed through intersections. This risky driving behavior reduces the ride’s comfort and diminishes the time for drivers to observe and evaluate the surrounding environment, thereby increasing safety risks.

Considering the negative impacts of time anxiety on driving safety, researchers have been consistently working towards reducing or removing such anxiety in driving tasks. The primary strategies utilized involve emotion-focused therapy and cognitive therapy [10]. Emotion-focused therapy is an external intervention that helps reduce drivers’ anxiety through external diversions like calming music, which can enhance drivers’ focus and alleviate time anxiety [11]. Conversely, cognitive therapy is an internal approach that address anxiety symptoms at the source by modifying the cognitive perspectives and thought patterns that trigger stress, with specific techniques including cognitive restructuring and cognitive-attention training. Among these, cognitive restructuring stands out as a crucial technique for easing time anxiety in driving tasks.

However, the current research related to cognitive therapy has not shown significant effectiveness in alleviating time anxiety during driving tasks. The main reason is that most studies only focused on the negative impacts of time anxiety on driving behavior and road safety risks, lacking in-depth observation and interpretation of changes in time-reduction performance during time anxiety driving tasks. The possibility of time-reduction is the most critical factor for drivers under time anxiety. Drivers often willingly take risks with dangerous driving behaviors if they believe it will shorten their travel time, even knowing that it might increase road safety risks. Therefore, the key to cognitive therapy for this issue lies in deepening the understanding of time performance in time anxiety driving tasks. People often believe that increasing driving speed and frequently changing lanes will reduce travel time, but this perception may not be entirely accurate. For example, in urban traffic jams, rushing through an intersection at a yellow light may only lead to increased waiting time at the next traffic light. Such driving experiences provide insight, compelling us to contemplate and broaden our understanding of the issue of time-saving performance in time-reduction-goal tasks:

(1) In urban roads, does the driving behavior of drivers with time-saving goals effectively reduce travel time? Furthermore, does the road segment type impact the amount of time saved?
(2) Can engaging in a task with the goal of reducing time cause drivers to feel anxious? If so, does anxiety result in alterations in driving behavior, consequently impacting travel time?

To explore these questions, the study conducted a within-subject controlled experiment in two conditions: one with time-reduction objectives and one without. The experiment involved gathering physiological measurements like heart rate and skin conductance, along with behavioral data such as longitudinal acceleration and frequency of lane changes, from a sample of 32 drivers. Additionally, the time taken for each trial was recorded. The main goal of the research was to evaluate and illustrate the actual influence of tasks related to reducing time on driving performance using time-related metrics, physiological parameters of drivers, and behavioral metrics of driving. The results were then translated into novel knowledge and integrated into a new survey to evaluate whether the participants resonated with the experimental results, thus confirming whether the experiment effectively altered the drivers’ perception of the matter.

## 2. Materials and methods

**Fig 1:**
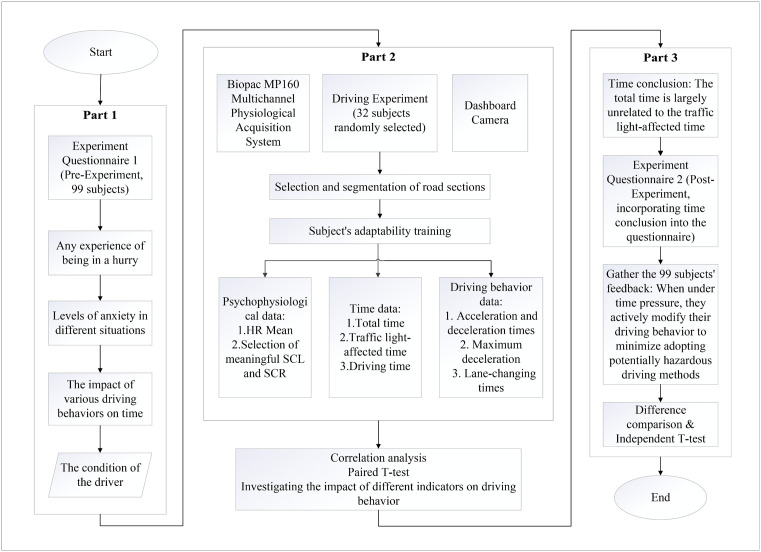
Research flow chart.

As shown in Fig 1, the study’s complete workflow consists of the following stages:

(1) A time anxiety questionnaire was designed and openly distributed on campus, targeting male individuals with significant driving experience (more than 10,000 kilometers driven) to participate in the survey. The questionnaire was developed by the authors, drawing on constructs discussed in previous research on time pressure and driver stress [12]. As a result, 99 valid responses were collected.
(2) Determine the designated road segment for the experiment, assess the road conditions on this road, and then complete the experimental design, which involves time to conduct experiment and defining tasks with or without time constraints.
(3) 32 subjects were randomly chosen from the pool of 99 respondents who completed the valid time anxiety questionnaires to take part in the actual vehicle driving experiment.
(4) The driving experiment was carried out to gather data on driving behavior and psychophysiological responses from 32 participants in two different conditions: with and without time-reduction-goal tasks. And then an analysis will be conducted by comparing the two groups.
(5) A feedback survey was created by using comparative analysis outcomes. The 99 eligible participants from the original survey on time anxiety were notified about the experimental outcomes. Among these, 32 individuals were part of the real vehicle driving test, identified as the “experimental group,” while the remaining 67 individuals, who did not take part in the experiment, were labeled as the “social group.” Finally, a comparative evaluation of the feedback survey responses from both groups was carried out.

### 2.1. Questionnaire I: Survey on the perception of time anxiety

A survey was designed to explore the awareness levels of driving task with time anxiety of young male individuals on campus. The survey aimed to evaluate perceptions of time-induced anxiety through a series of questions. These questions included the following:

Q1: Have you experienced driving in a hurry on urban roads with traffic lights?

Q2: Did you perceive the waiting time at traffic lights to be longer when you were in a rush compared to usual?

Q3: Did you perceive the vehicles in front of you moving slowly when driving in a hurry?

Q4: Did you frequently check the time when encountering traffic congestion?

Q5: Which of the following behaviors did you exhibit when driving in such situations? (Accelerating to increase speed whenever possible, frequently changing lanes to overtake, proceeding through the intersection on a flashing green (or yellow) light, cutting the queue, or other behaviors)

Q6: Do you believe that adopting the aforementioned driving behaviors on routes with multiple traffic lights can effectively reduce travel time?

Q7: Do you believe that adopting the aforementioned driving behaviors on traffic light-affected sections (defined as the area within 50 meters of an intersection, spanning from 50 meters before to 50 meters after the lights, as specified in “The Regulation on the Implementation of the Law of the People’s Republic of China on Road Traffic Safety”) can effectively reduce waiting time?

Q8: Do you believe that adopting the aforementioned driving behaviors on non-traffic light-affected sections (the area between 50 meters after one set of lights and 50 meters before the next set) can effectively reduce driving time?

Q9: Do you believe the driving behavior resulting from rushing impacts road safety?

**Fig 2:**
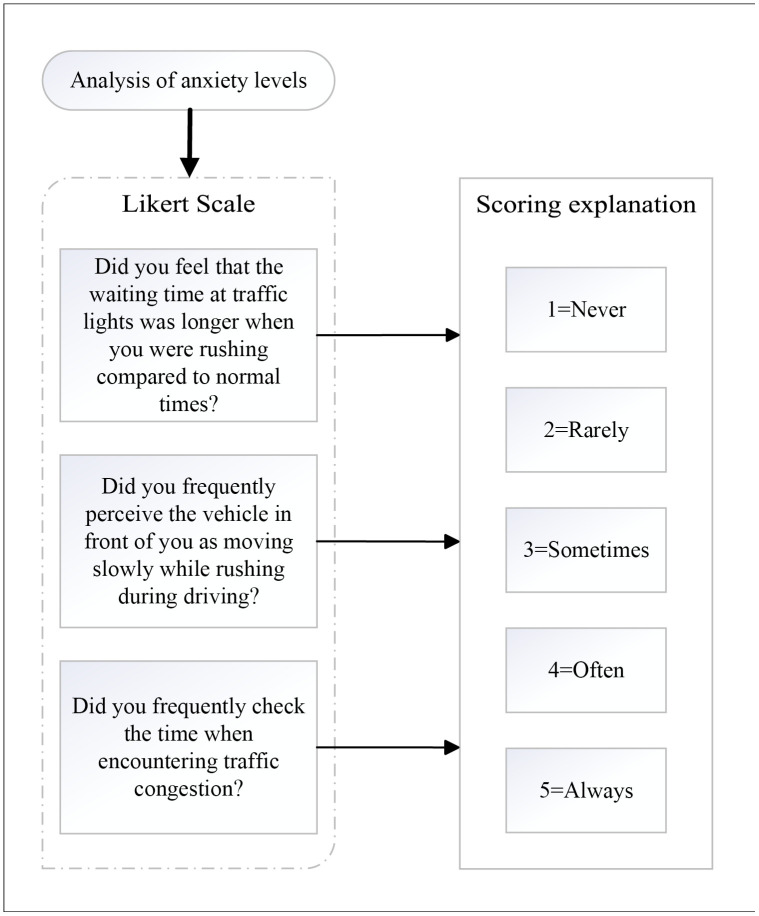
Anxiety level question set.

In the questions mentioned above, Q2-Q4 are the anxiety level question set, using a Likert scale (1 = Never; 2 = Rarely; 3 = Sometimes; 4 = Often; 5 = Always) to reflect the respondents’ level of anxiety under the respective conditions (Fig 2);

In Q7 and Q8, the road segments were categorized into traffic light-affected segments and non-traffic light-affected segments, to assess participants’ comprehension of how travel time on different road segments relates to total time.For Q9, Likert scale (1 = Definitely will; 2 = Very likely; 3 = Uncertain; 4 = Probably not; 5 = Definitely not) was used to explore respondents’ perceptions regarding the safety of hazardous driving behavior.

### 2.2. Experiment design

#### 2.2.1. Experiment route.

**Fig 3:**
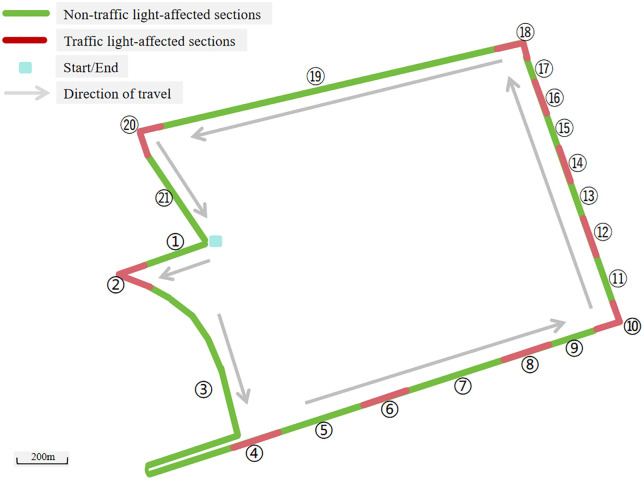
Driving experiment route.

The selected experiment route for this study was an urban road encircling the campus, as shown in Fig 3. The total length of this typical urban road was 7.1 kilometers, with 10 traffic signals along the way (4 for left turns and 6 for going straight). Consequently, the experimental driving route was segmented into 21 parts, comprising 10 traffic light-affected segments and 11 non-traffic light-affected segments.

#### 2.2.2. Experiment equipment.

The study utilized a NISSAN ALTIMA 2020 vehicle with dimensions of 4901*1850*1447 mm and a 2.0L engine generating 156 horsepower. The experimental setup included a Biopac MP160 Multichannel Physiological Acquisition System (made in the USA) and a 360 dashboard camera model G580 Pro.

**Fig 4:**
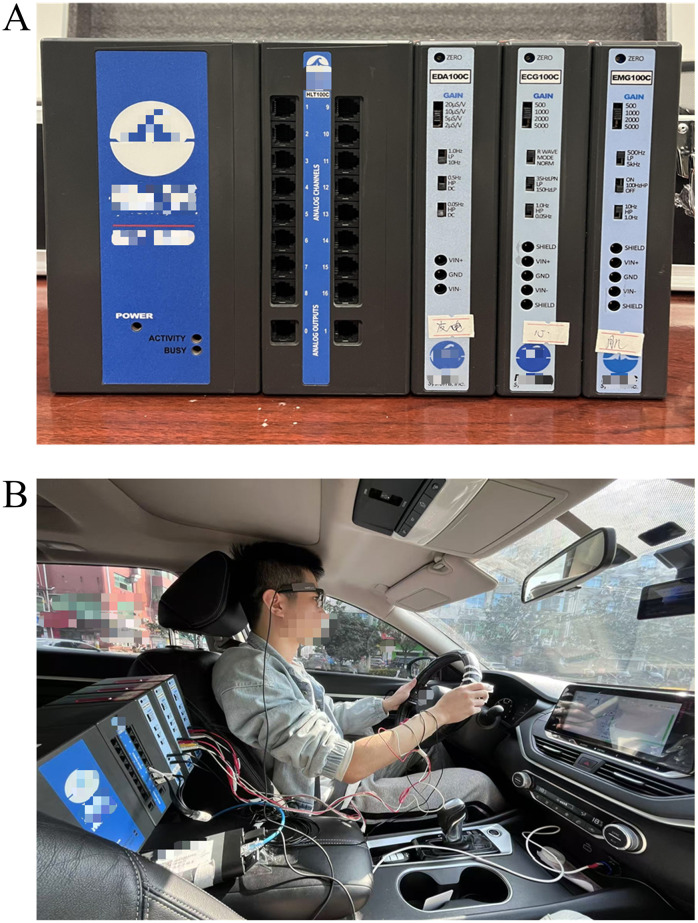
Experiment equipment. **(A)** The Experimental device BIOPAC MP-160, **(B)** The subject wore the devices. The subject has provided written consent for the publication of this image.

The data collection and analysis system employed in the experiment was the BIOPAC MP160 (Fig 4A), which is equipped with 16 channels and comes with the professional analysis software AcqKnowledge (Fig 4B). The X, Y, and Z ports of the accelerometer were connected to individual signal channels, and two amplifier modules (EDA100C and ECG100C) were also connected to separate signal channels. Data was collected at a sampling rate of 2000Hz. The accelerometer measured real-time acceleration in the direction of the vehicle’s motion. The EDA100C module recorded electrodermal activity data with settings of 5μS/V GAIN amplification and high-pass low-pass filters at 10 Hz, DC, DC. The ECG100C module collected electrocardiogram data with settings of 1000 GAIN amplification and high-pass low-pass filters at NORM, 35HzLPN, 1.0 Hz.

The 360 dashboard camera, featuring an SC501AI image sensor, captured front-view videos of the vehicle at a resolution of 2880 x 1620. It recorded timestamps and the vehicle’s driving speed simultaneously at 30fps.

#### 2.2.3. Subjects.

Following a thorough review of the answers of Questionnaire I, 32 healthy male individuals, aged 20–28 with optimal eyesight, were selected randomly to take part in the study. All participants held a C1 driver’s license and had accumulated over 10,000 kilometers of driving experience. Participants for this study were prospectively recruited between December 26, 2023, and January 10, 2024. All participants provided written informed consent prior to their involvement in the study.

#### 2.2.4. Experiment scenarios.

In order to investigate the influence of time-related anxiety on driving behavior, the experiment was designed with two distinct scenarios. In the first scenario, participants were not under any time pressure and were free to drive as they normally would. The second scenario, however, involved tasks aimed at reducing driving time, with an added incentive: each of the top five drivers who managed to have the least total driving time would be given a prize of 500 CNY. Each participant completed both driving scenarios along the designated experimental route, typically finishing the first scenario on the initial day of participation and the second scenario at the same time on the following day.

#### 2.2.5. Experiment procedure.

The experimental procedure is outlined as follows:

(1) Preparatory Phase: Prior to commencing the experiment, participants were given sufficient time (minimum of 10 minutes) to adjust the seat and mirrors to their comfort and get acquainted with operating the vehicle’s accelerator, parking brake, steering wheel, mirrors, and seat. Following this, the researcher provided the participants with an overview of the experimental requirements, guided them through the navigation, connected them to the physiological devices, and informed them that they could initiate the experiment once they felt comfortable with the equipment. Meanwhile, data collectors verified the proper functioning of the connections between the physiological instruments and computer software, and monitored the data collection and transmission process.
(2) Instrument Testing and Driving Commencement: Once the participant was ready, a brief assessment of the instruments was carried out. Upon achieving stable recordings from the instruments, the researcher indicated the start of the driving task and data recording. If the participant experienced any discomfort, the experiment was immediately stopped. Throughout the experiment, specific events such as the start/end points and the status of traffic lights within a 50-meter range were logged in the AcqKnowledge 5.0 software (e.g., Event 1 for start/end using the F1 key; Event 2 for red light with F2 key; Event 3 for green light with F3 key), aiding in the categorization of experimental data related to different road segments. Additionally, a reminder about upcoming traffic lights was programmed at the 7th segment, where researchers verbally notified drivers about the approaching traffic light-controlled intersection to evaluate variations in skin conductance response (SCR) under different stimulus conditions.
(3) Post-Driving Rest and Conclusion: After each driving task, participants rested for at least 3 minutes. The total duration of the test for each participant ranged from 30 to 35 minutes. After the rest period, participants removed the experimental gear, exited the vehicle, and concluded their participation in the experiment.

### 2.3. Questionnaire II: Cognitive feedback questionnaire

A cognitive feedback questionnaire was developed to inform the 32 subjects and the other 67 respondents who participated in Questionnaire I, forming the corresponding experimental group and social group. The questionnaire included the following questions:

Q1: Did the experimental results change your understanding of these tasks?

Q2: What decisions will you opt for when facing time-reduction-goal tasks in the future? (Persist in participating in the time-reduction-goal driving behavior, slightly decrease the occurrence of time-reduction-goal driving behavior, abstain from embracing time-reduction-goal driving behavior unless essential (excluding special emergencies), consistently stick to the usual driving behavior)

For Q1, a Likert scale (1 = Completely as anticipated, 2=As anticipated, 3 = Mostly as anticipated, 4 = Not quite as anticipated, 5 = Completely surprising) was used to investigate the impact of cognitive intervention on the experimental outcomes across the two distinct groups.

### 2.4. Experiment data preprocessing

The experimental team organized and analyzed the collected data. For the 32 participants, two driving conditions yielded a total of 64 datasets, which were categorized into time data, driving behavior data, and physiological data. These three categories and their corresponding metrics are detailed below.

Roadside markers were placed 50 meters before and after each traffic light to define the start and end points of 21 road segments. Using dashboard camera footage, timestamps and corresponding instantaneous speeds were extracted. Data were sampled at 32 Hz, and the duration of each segment was calculated by subtracting the entry time from the exit time across the marked locations.

To record the status of traffic lights at the start of each segment, Biopac hotkeys F2 and F3 were used. AcqKnowledge 5.0 software was then employed to segment the physiological data according to the traffic light event markers. Heart rate (HR) and skin conductance level (SCL) data were extracted for the full route and for the 10 segments affected by traffic lights. HR data, derived from ECG signals, were processed using the software’s “find rate” function and averaged by segment. SCL data were analyzed to identify skin conductance responses specifically linked to red-light events.

## 3. Results

### 3.1. Survey questionnaire results

**Fig 5:**
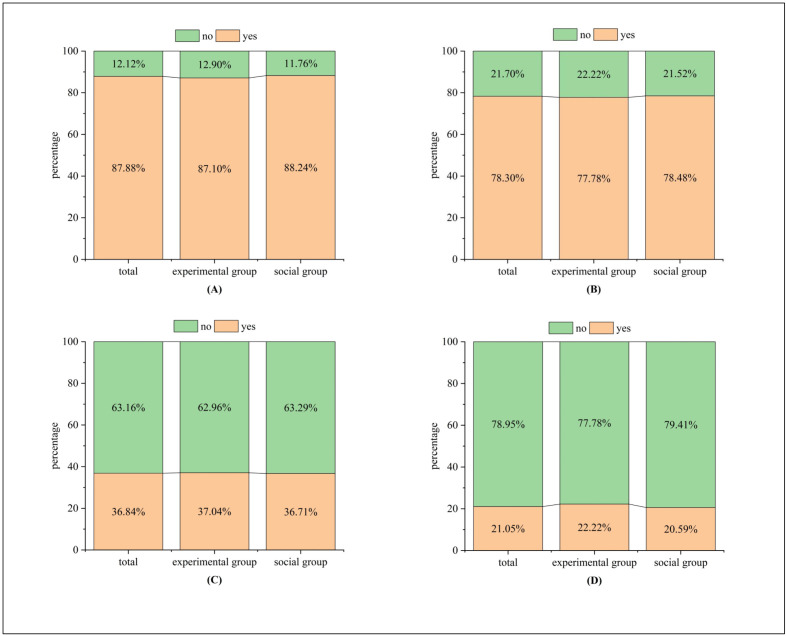
Time anxiety questionnaire percentage stacked bar chart. **(A)** Q1, **(B)** Q6, **(C)** Q7, **(D)** Q8.

In order to fully comprehend how drivers behave when feeling time anxiety and the implications of the experimental outcomes, the data obtained from 99 valid surveys (32 from the experimental group and 67 from the social group) were examined. According to the results presented in Fig 5A, 87.10% of drivers in the experimental group admitted to rushing on urban roads with traffic signals, a percentage closely matched by 88.24% in the social group. In total, 87.88% of all participants displayed rushing behavior on urban roads with traffic lights, demonstrating consistency between the experimental and social groups.

A survey was conducted to examine the driving habits of two groups in situations where time was limited. The results indicated that drivers exhibited different behaviors when in a hurry, with the most prevalent one being speeding up to enhance their velocity. As shown in Fig 5B, about 78% of participants in both the experimental group (22.22% no, 77.78% yes) and the social group (21.52% no, 78.48% yes) thought that using a faster driving approach could help cut down the overall travel time on routes with numerous traffic signals.

The survey results about total time(TT) can be divided into two parts: the traffic light-affected time(TLT) and the non-traffic light-affected time(NTLT), and these two results indicate: participants across all three groups perceive the TT as a crucial factor in reducing overall time consumption. The total percentage is 36.84%, with the experimental group at 37.04% and the social group at 36.71% (refer to Fig 5C). Additionally, a significant number of respondents believe that adjusting driving behavior can effectively reduce driving time, with an overall proportion of 71.58%. In this aspect, the experimental group represents 70.37%, and the social group accounts for 72.15% (refer to Fig 5D).

**Fig 6:**
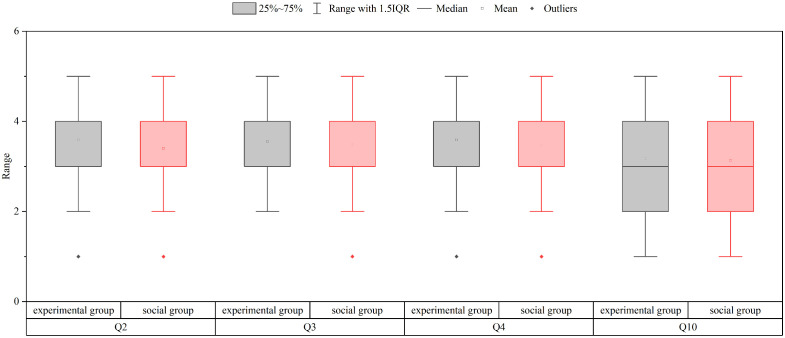
Results of the questionnaire i likert scale questions.

Based on the result from the anxiety assessment questionnaire (consisting of three Likert 5-point scale questions) in various situations, both the experimental group and the social group obtained similarly high scores, indicating that participants in both groups experienced a significant level of anxiety when confronted with driving pressure (depicted in Fig 6). Subsequent result of analysis indicated that there was no notable distinction in the scores of the two groups for each scale question. This was supported by the results for Q2 (F2 = 0.585, *p*2 = 0.446), Q3 (F3 = 0.666, *p*3 = 0.416), and Q4 (F4 = 0.400, *p*4 = 0.528), confirming the likeness in their perceptions of anxiety.

For Question 9, concerning the influence of hurried driving behaviors on road safety, higher scores indicated that drivers had a more positive outlook on how these behaviors may affect safety. Both groups of respondents displayed an optimistic view regarding the impact of rushing-induced driving behaviors on road safety, with no notable difference observed (*p* = 0.152) (As shown in Fig 6).

### 3.2. Real-vehicle experiment results

The results from Questionnaire I also confirmed that the characteristics of the 32 randomly selected subjects matched the overall properties of the 99 respondents’ sample, that means the experimental outcomes derived from the 32 chosen participants could reflect the situation of all samples. In this driving experiment, we collected experimental data from 32 subjects under two different scenarios. An in-depth analysis was conducted on the subjects’ time data, driving behavior data, and physiological data. To dissect the performance differences of subjects under scenarios with and without time-reduction-goal tasks, we performed paired T-tests on the various indicators of the 32 subjects across the two distinct scenarios. However, after a stringent data screening process, one subject’s data that did not meet the analysis criteria was excluded. Therefore, we ultimately chose 31 valid sample data subjects for subsequent research and analysis.

#### 3.2.1. Time data.

**Fig 7:**
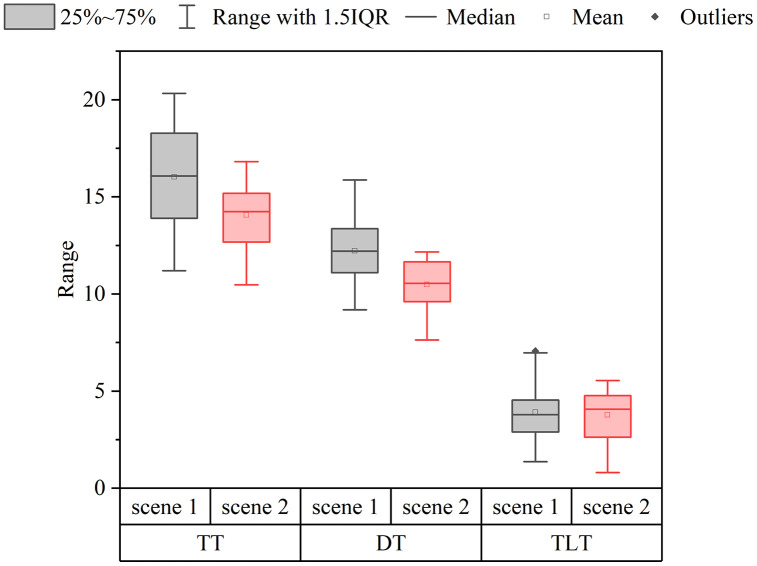
Time data results.

Table 1 shows the statistical analysis result on driving time and behavior indexes in the two scenarios, and the data distribution is shown in Fig 7. By performing a paired t-test on the data collected from the two scenarios, we found that there was a significant difference between the total time (TT) of the two scenarios (t = 4.792, *p* < 0.01), with the TT of Scenario 2 (Mean = 14.062, SD = 1.665) being on average 1.96 minutes less than the TT of Scenario 1 (Mean = 16.023, SD = 2.454). There was also a significant difference between the non-traffic light-affected time (NTLT) of the two scenarios (t = 7.948, *p* < 0.01),with Scenario 2 NTLT (Mean = 10.481, SD = 1.155) averaging 1.73 minutes less than Scenario 1 NTLT (Mean = 12.212, SD = 1.574). However, no significant difference was demonstrated between Scenario 1 (Mean = 3.912, SD = 1.456) and Scenario 2 (Mean = 3.768, SD = 1.2241) in terms of the traffic light-affected time (TLT) (t = 0.539, *p* = 0.594).

**Table 1:** Data types and definitions.

Data Type	Indicator Names	Indicator Definition	Units
**Time Data**	total time, TT	Total time taken by the driver to complete the experimental route	min
	traffic light-affected time, TLT	Total time taken by the driver to complete the traffic light-affected segments of the experimental route	min
	non-traffic light-affected time, NTLT	Total time taken by the driver to complete the non-traffic light-affected segments of the experimental route	min
	total time difference, TTD	Difference in total time taken by the driver between Scenario 2 and Scenario 1 to complete the experimental route	min
	non-traffic light-affected time difference, NTLTD	Difference in time taken by the driver between Scenario 2 and Scenario 1 to complete the non-traffic light-affected segments of the experimental route	min
	traffic light-affected time difference, TLTD	Difference in time taken by the driver between Scenario 2 and Scenario 1 to complete the traffic light-affected segments of the experimental route	min
**Driving Behavior Data**	acceleration and deceleration times, ADT	Number of accelerations and decelerations by the driver within the total time	time
	maximum deceleration, MD	Maximum deceleration by the driver during the total time	m/s^2^
	lane-changing times, LCT	Number of lane changes by the driver within the total time	time
	acceleration and deceleration times difference, ADTD	Difference in the number of accelerations and decelerations by the driver between Scenario 2 and Scenario 1 within the total time	time
	maximum deceleration difference, MDD	Difference in the maximum deceleration by the driver between Scenario 2 and Scenario 1 during the total time	m/s^2^
	lane-changing times difference, LCTD	Difference in the number of lane changes by the driver between Scenario 2 and Scenario 1 within the total time	time
**Physiological Data**	heart rate, HR	Average heart rate of the driver while completing the experimental route	beats per minute
	skin conductance level, SCL	Average skin conductance level of the driver throughout the experimental route	µS
	skin conductance response, SCR	Change in skin conductance response of the driver when encountering a red traffic light reminder during the driving process	µS
	HR difference, HRD	Difference in heart rate of the driver between Scenario 2 and Scenario 1 when completing the experimental route	beats per minute
	SCL difference, SCLD	Difference in skin conductance level of the driver between Scenario 2 and Scenario 1 when completing the experimental route	µS
	SCR difference, SCRD	Difference in the change in skin conductance response of the driver when encountering a red traffic light reminder between Scenario 2 and Scenario 1 during the driving process	µS

To gain a deeper understanding of the underlying reasons for this occurrence and to explore the possible hazards involved, we conducted an analysis of the total time (TT), the traffic light-affected time (TLT), and the non-traffic light-affected time (NTLT) values for the 21 road segments in Scenarios 1 and 2. We compared the outcomes presented to delve into the primary road segments contributing to time variations, as shown in Table 2. Our findings revealed that out of the 21 road segments, five sections exhibited notable variances (*p* < 0.01) in NTLT, specifically sections 3, 5, 7, 19, and 21, all of which are the non-traffic light-affected segments. Conversely, there were no significant differences (*p* > 0.05) in the TLT values for any of the 10 traffic light-affected segments in both scenarios.

**Table 2:** Paired t-test results of 21 road segments.

indicators	NTLT						indicators	TLT					
	Mean		SD		t	*p*		Mean		SD		t	*p*
	Scene1	Scene2	Scene1	Scene2				Scene1	Scene2	Scene1	Scene2		
Segment1	0.198	0.130	0.184	0.041	2.024	0.052*	Segment2 (traffic light-affected)	1.013	1.092	0.501	0.522	−0.573	0.571
Segment3	2.888	2.337	0.612	0.342	5.683	0.000***	Segment4 (traffic light-affected)	0.827	0.698	0.538	0.401	1.532	0.136
Segment5	0.436	0.353	0.085	0.070	5.446	0.000***	Segment6 (traffic light-affected)	0.759	0.795	0.470	0.462	−0.357	0.724
Segment7	0.518	0.420	0.176	0.083	3.853	0.001***	Segment8 (traffic light-affected)	0.587	0.546	0.360	0.289	0.484	0.633
Segment9	0.466	0.398	0.191	0.194	2.068	0.047**	Segment10 (traffic light-affected)	1.239	0.982	0.927	0.643	1.529	0.137
Segment11	0.617	0.575	0.162	0.121	1.315	0.199	Segment12 (traffic light-affected)	0.468	0.435	0.322	0.290	0.418	0.679
Segment13	0.352	0.320	0.074	0.131	1.324	0.196	Segment14 (traffic light-affected)	0.563	0.515	0.363	0.366	0.494	0.625
Segment15	0.101	0.095	0.017	0.021	1.647	0.110	Segment16 (traffic light-affected)	0.475	0.443	0.208	0.241	0.532	0.599
Segment17	0.083	0.074	0.019	0.021	1.858	0.073*	Segment18 (traffic light-affected)	1.020	1.016	0.482	0.397	0.034	0.973
Segment19	1.702	1.453	0.259	0.144	6.236	0.000***	Segment20 (traffic light-affected)	1.305	1.069	0.461	0.540	1.978	0.057*
Segment21	0.501	0.403	0.084	0.066	6.277	0.000***	–	–	–	–	–	–	–

***P < 0.01,**P < 0.05,*P < 0.1.

#### 3.2.2. Driving behavior data.

There are three types of driving behavior data, namely the number of acceleration and deceleration times (ADT), the lane-changing times (LCT), and the maximum deceleration (MD).

As shown in Table 3, after conducting a paired t-test on the data collected from the two situations, it was found that there was a highly significant difference in the number of ADT. This metric refers to the count of acceleration and deceleration occurring when the vehicle is moving straight ahead, between the two scenarios (t = −3.074, *p* < 0.01). In Scenario 2 (Mean = 14.774, SD = 4.279), it exhibited an increase of 2.29 times compared to Scenario 1 (Mean = 12.484, SD = 4.711). Additionally, a noticeable disparity was observed in the LCT carried out by drivers in the two scenarios (t = −2.804, *p* < 0.01), with a significant uptick in the number of LCT in Scenario 2 (Mean = 10.194, SD = 3.188) compared to Scenario 1 (Mean = 8.516, SD = 2.827). However, there was no significant difference in the driver’s MD, which indicates the maximum braking deceleration, between the two scenarios (t = −1.021, *p* = 0.315). The MD in Scenario 2 (Mean = 4.495, SD = 1.2) only increased by 0.354m/s2 in comparison to the MD in Scenario 1 (Mean = 4.141, SD = 1.556).

**Table 3:** Paired t-test results of time data, driving behavior data and physiological data.

Variable name	Scene 1					Scene 2					t	*p*
	Number	Max	Min	Mean	SD	Number	Max	Min	Mean	SD		
**TT**	31	20.33	11.2	16.023	2.454	31	16.82	10.47	14.062	1.665	4.792	0.000***
**TLT**	31	7.06	1.37	3.912	1.456	31	5.54	0.8	3.768	1.241	0.539	0.594
**NTLT**	31	15.87	9.19	12.212	1.574	31	12.17	7.63	10.481	1.155	7.948	0.000***
**LCT**	31	15	4	8.516	2.827	31	18	4	10.194	3.188	−2.804	0.009***
**MD**	31	8.585	1.509	4.141	1.556	31	7.242	2.607	4.495	1.2	−1.021	0.315
**ADT**	31	22	6	12.484	4.711	31	23	3	14.774	4.279	−3.074	0.004***
**HR**	31	117.77	59.765	94.017	12.105	31	121.373	58.612	95.195	13.582	−1.37	0.181
**SCL**	31	20.779	0.016	5.282	5.243	31	15.188	0.411	5.534	3.642	−0.361	0.721
**SCR**	31	8.655	0.061	1.566	2.386	29	15.759	0.021	1.602	2.863	−0.134	0.895

***P < 0.01,**P < 0.05,*P < 0.1.

#### 3.2.3. Physiological data.

The three types of physiological data comprised electrodermal and electrocardiographic measurements. Specifically, electrocardiographic data included skin conductance level (SCL) and skin conductance response (SCR), while electrocardiographic data was heart rate (HR).

As shown in Table 1, by carrying out a pair t-test of the driver’s SCL during the two driving scenarios, we found that the average SCL value between the two scenarios (t = −0.361, *p* = 0.721) was not significantly different, and the average HR value between the two scenarios was not significantly different (t = −1.37, *p* = 0.181). In addition, when we analyzed the size of the driver’s SCR in two scenarios, when red light warning events occurred, we found no significant differences in SCR values between the two scenarios (t = −0.134, *p* = 0.895).

#### 3.2.4. Correlation test.

**Fig 8:**
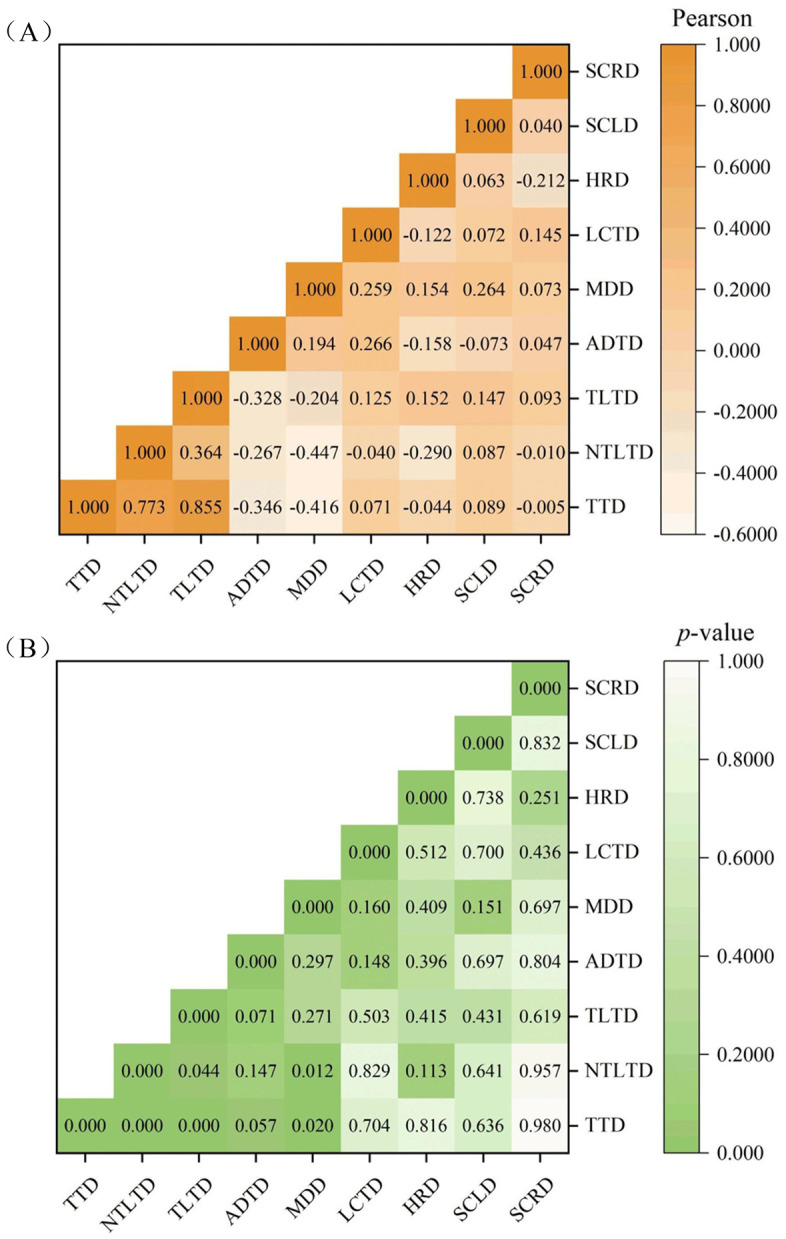
Correlation analysis of discrepant data with time. **(A)** The Pearson correlation heat map, **(B)** The *p*-value heat map.

To further explore the potential connections between differences in driving behavior data, differences in time data, and differences in physiological data, we conducted correlation tests to obtain a more comprehensive understanding. As shown in Fig 8, after in-depth analysis of the data, the non-traffic light-affected time difference (NTLTD) (Pearson = 0.773, *p* = 0.01) and the traffic light-affected time difference (TLTD) (Pearson = 0.855, *p* = 0.01) had significant positive correlations with the total time difference (TTD), which means that when NTLTD and TLTD decreased, the corresponding TTD decreased. Both the acceleration and deceleration times difference (ADTD) (Pearson = −0.346, *p* = 0.1) and the maximum deceleration difference (MDD) (Pearson = −0.416, *p* = 0.05) were negatively related to TTD. Specifically, when ADTD and MDD increased, the value of the TTD would be smaller. Besides, ADTD values had significant negative correlations (Pearson = −0.328, *p* = 0.1) with TLTD values, MDD values had significant negative correlations (Pearson = −0.447, *p* = 0.05) with NTLTD. Furthermore, the lane-changing times difference (LCTD) did not have a significant correlation with TTD, TLTD and NTLTD, and the heart rate difference (HRD), the skin conductance level difference (SCLD), and the skin conductance response difference (SCRD) did not have a significant correlation with TTD or NTLTD.

### 3.3. Cognitive feedback questionnaire result

**Fig 9:**
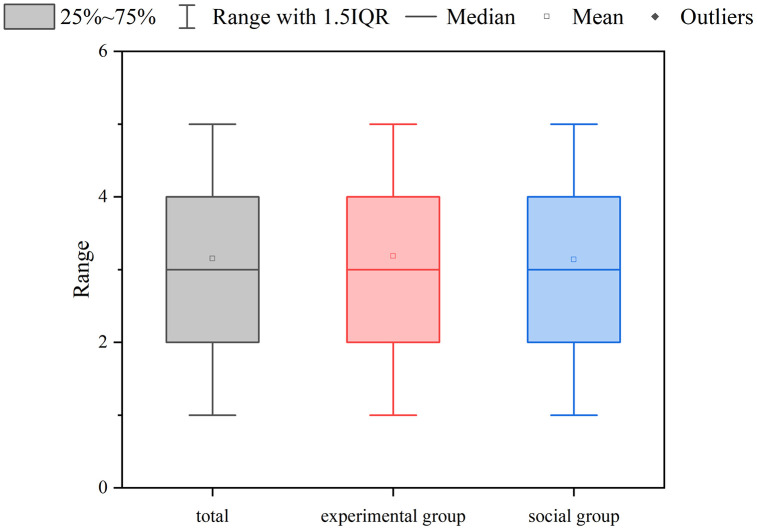
Result of cognitive feedback questionnaire likert scale questions.

The Cognitive Feedback Questionnaire was given to the participants after they were informed of the experimental outcomes, to study whether their cognitive shifts following the realization that aggressive driving behavior did not effectively reduce the traffic light-affected time (TLT). To assess the degree to which participants anticipated these experimental findings, we analyzed the statistical outcomes of the Likert scale inquiries, depicted in Fig 9. The analysis result revealed that there was no significant difference in scores between the two groups (*p* = 0.398): the experimental group had an average score of 3.32, and the social group had an average score of 3.29. In general, the experimental results indicated a deviation from the initial understanding of the participants.

**Fig 10:**
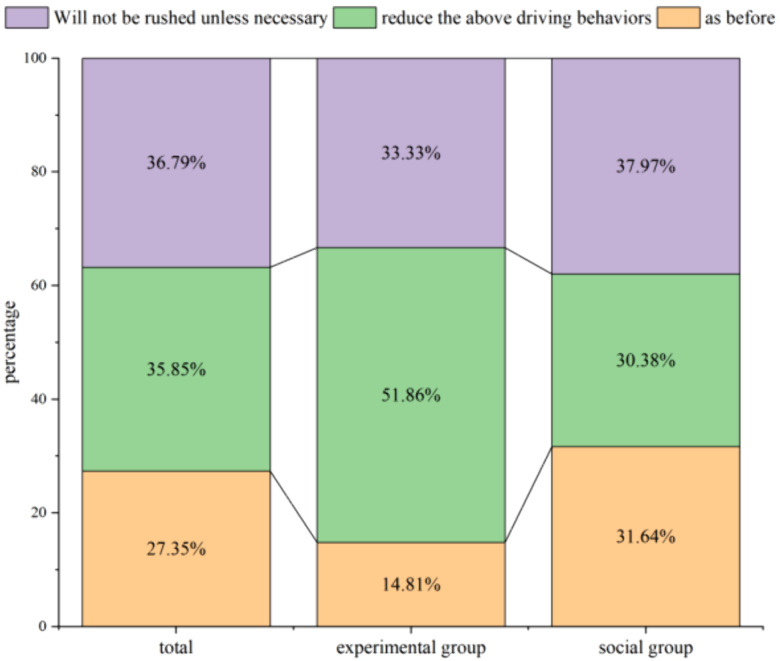
Result of cognitive feedback questionnaire bar chart.

It is important to note that, following the understanding of the various cognitive knowledge derived from the experimental findings, 33.33% of the people in the experimental group chose not to drive in a hurry unless necessary, and 51.86% said they would appropriately reduce aggressive driving behaviors; 37.97% of the drivers in the social group said they would try to avoid taking aggressive driving behaviors. And 21.48% fewer people chose to appropriately reduce the above-mentioned driving behaviors than the experimental group. Of course, in the experimental group and social group, 14.81% and 31.64% respectively said they would stick to their original driving habits (Fig 10).

## 4. Discussion

Using within-subject control tests under two real-world driving scenarios—with and without a time-reduction-goal task—the study found that time incentives encouraged drivers to complete the route more quickly. In the time-reduction-goal condition, the total travel time (TT) was reduced by an average of 1.96 minutes compared to the control condition. Howerver, when the 21 road segments were categorized into traffic light-affected and non-traffic light-affected segments, significant time differences were observed in 9 segments. Of these, 8 were non-traffic light-affected (38% of all segments), while only 1 was traffic light-affected (4.7%), as shown in Table 3. This indicates that the observed reduction in TT was primarily driven by decreased non-traffic light-affected time (NTLT), with minimal effect on traffic light-affected time (TLT). This is likely because signal-controlled intersections impose fixed delays that drivers cannot bypass through behavioral changes. This result is different from the traditional perception of drivers, which was pointed out by Fuller’s survey of 1,005 drivers in the UK that most drivers believed that a high starting speed at traffic lights could more effectively save travel time [13]. However, in practice, aggressive acceleration before red lights often results in longer waiting times at the next intersection, offering little actual time-saving. The results of this experiment give rise to another interesting understanding of driving experience: the drivers’ speed in time-reduction-goal tasks may increase waiting time at the next intersection of traffic lights, but it may not increase waiting time at specific traffic lights. This is because each stoplight time on the journey is already set in advance and the driver may speed up through the stoplight and change the status of the stops encountered throughout the journey is low.

As shown in the above analysis, the reduction in total time (TT) during time-reduction-goal tasks was strongly associated with an increase in acceleration and deceleration times (ADT) and a higher absolute value of maximum deceleration (MD). These changes indicate that drivers exhibited more aggressive driving behavior under time-reduction-goal conditions.In addition to time pressure, the design of the time-reduction-goal task itself—particularly in Task 2—may have further contributed to aggressive behavior. Unlike general urgency, the competitive, reward-based framing of the task likely activated goal-directed motivation, encouraging drivers to adopt more assertive strategies to outperform perceived benchmarks..This finding aligns with previous studies [14,15], which reported that drivers under time pressure tend to adjust their speed and change lanes to reduce TT [16,17], most commonly by increasing their speed [18,19]. However, such behavior raises the risk of unsafe driving, potentially leading to traffic accidents or congestion [9,20]. Unlike earlier observations, this study did not find evidence that lane-changing contributed to more efficient driving. This discrepancy may be attributed to the low traffic volume on the test road, where drivers had limited opportunities to overtake or perform lane changes during the experiment. Moreover, under such low-traffic conditions, the risks typically associated with lane-changing—such as sudden braking, unsafe gaps, or vehicle conflicts—were likely diminished. As a result, participants may have engaged in more frequent lane changes without experiencing the usual level of perceived risk or tension [21,22,23].

However, the experiment found no evidence that the time-reduction-goal task induced psychological anxiety in drivers. Across both scenarios, there were no significant changes in average heart rate (HR), nor were there notable differences in skin conductance response (SCR) when drivers encountered the same stimulus events. Correlation analysis also indicated no significant relationship between physiological or psychological states and driving behavior. Although some researchers have suggested a close link between time anxiety and aggressive driving behavior [12], the findings here diverge. For instance, Peer [17] reported that drivers may experience greater time-related stress due to external factors such as traffic complexity and tight schedules. These conditions can provoke emotional responses that influence driving behavior and decision-making. From a physiological standpoint, metrics such as HR, skin conductance level (SCL), and SCR are commonly used to objectively reflect psychological responses to stress or anxiety [24]. The present findings may be explained by two factors. First, under time-reduction-goal tasks, drivers may have demonstrated stronger emotional regulation and psychological adaptability, along with confidence in managing the risks associated with aggressive driving. Second, the time-reduction-goal in this experiment was framed as a reward-based incentive, which differs fundamentally from anxiety typically induced by punishment mechanisms [18]. In real-world contexts—such as rushing to catch a train or flight—time pressure often carries the threat of loss, making the psychological burden far greater than in reward-driven tasks like those used here. Third, the behavioral responses observed under time incentives may have been more instrumental than emotional—driven by task-focused motivation rather than emotional arousal [25]. In this context, drivers may have adopted a strategic approach to maximize rewards, engaging in faster or more frequent maneuvers while maintaining internal composure. This interpretation is supported by the lack of strong correlations between physiological signals and behavioral indicators.

Based on both the questionnaire and time-comparison test results, it is evident that drivers facing time-reduction-goal tasks exhibited more aggressive driving behavior than those without such tasks, despite being aware of the potential safety risks. These behaviors increased personal driving risk [26], although they did not violate any traffic laws or regulations [27]. For traffic managers, these invisible risks are particularly difficult to monitor and prevent, posing a significant challenge to improving road safety [28–30].

According to the questionnaire, many drivers believed that aggressive behavior could shorten travel time, especially the traffic light-affected time (TLT), when under time-reduction pressure—an expectation not supported by experimental data. To address this cognitive bias, we explored physiological interventions and cognitive restructuring strategies [31] to influence driver behavior under time-reduction-goal tasks. The experimental findings were shared with all 99 participants from the initial survey (Question I), followed by a cognitive feedback questionnaire to assess changes in perception between experimental and non-experimental groups.

Feedback results showed that most drivers accepted the study’s conclusion that aggressive driving did not reduce TLT and became more aware of time anxiety in driving tasks. Notably, a larger proportion of experimental participants indicated they would reduce or avoid aggressive behavior in future time-reduction-goal scenarios. This shift may stem from a deeper understanding of how seemingly time-saving behaviors can [32], in fact, decrease efficiency and introduce hidden dangers [33–35]. These findings support the effectiveness of cognitive feedback in reducing risk and modifying behavior in time-pressured driving contexts. This may be because experimental participants received personalized, data-driven feedback based on their own driving performance, making the risks more tangible. In contrast, the social norm group received general behavioral recommendations, which may have appeared less relevant or persuasive. Such individualized feedback likely enhanced behavioral awareness and motivation for change.

These findings demonstrate the potential of cognitive restructuring to promote safer driving decisions under time pressure. This aligns with the Theory of Planned Behavior (TPB) [36], which suggests that behavioral change is influenced by attitudes, subjective norms, and perceived behavioral control. In this study, individualized feedback may have reshaped drivers’ attitudes toward aggressive behavior by revealing its limited time-saving effect and increased risk. By altering these cognitive appraisals, the intervention likely enhanced drivers’ intention to change, thereby facilitating actual behavioral adjustment. This theoretical linkage supports the use of targeted feedback as an effective behavior-change strategy in traffic safety interventions.

### 4.1. Limitations

This study recruited only young male drivers with more than 10,000 km of driving experience, most of whom were university students. This limited sample scope excluded female drivers, non-student populations, and individuals with less driving experience. Such demographic and experiential constraints may introduce sample bias and reduce the generalizability of the findings. Future studies should include participants with more diverse backgrounds in terms of age, gender, occupation, and driving experience to assess whether the behavioral and physiological responses observed here apply to broader driver populations.

Secondly, the time anxiety of the experiment was introduced mainly through reward mechanisms, which are slightly different from real situations with clear time constraints, such as bus entry. In addition, due to limited sample data, it was difficult to establish a quantitative model for reducing driving behaviour over time in our study, which also questioned the reliability of the results. In future research, more sample expansion and analysis of factors are required to ensure more comprehensive and reliable conclusions.

## 5. Conclusion

This study analyzed the impact of time-saving objectives on urban driving behavior and examined how experimental results reshaped drivers’ perceptions, particularly in traffic light-affected segments. To address three key issues related to time anxiety and travel time, we conducted naturalistic driving experiments and distributed surveys along campus-area routes to assess drivers’ psychological and behavioral responses. The results showed that time-reduction goals led to more aggressive driving—such as speeding and frequent lane changes—which effectively shortened travel time in non-traffic light-affected segments but had little impact in segments influenced by traffic lights. The incentivized task did not significantly increase psychological anxiety but did increase risky behavior.

The incentivized time-saving task does not significantly increase the psychological anxiety of the driver, but may lead to more aggressive driving behaviour, increasing the risk of road safety. The study proposed the use of cognitive restructuring psychotherapy to restore the drivers’ cognition in these driving tasks to address the problem of traffic safety arising from the lack of external regulation measures. The real-world experimental results demonstrating that driving behaviour cannot significantly reduce time in traffic-light affected segments were widely accepted by drivers and yielded positive psychological interventions. This confirms the effectiveness of cognitive restructuring and intrinsic guidance to induce changes in drivers’ awareness and to improve road safety at the social level. The conclusions of the study provide valuable insights for road safety management and education and contribute to the orderly development of the road safety environment.

## Supporting information

S1 File Provides the information related to Research flow, driving experiment route, experiment equipment, questionnaire data and various data collected from driving experiment. (XLSX)
